# A neonatal mouse model of meconium peritonitis generated using human meconium slurry

**DOI:** 10.1038/s41390-024-03470-3

**Published:** 2024-08-21

**Authors:** Mariko Ashina, Takumi Kido, Keisuke Shirai, Yu Masuda, Yukihito Imagawa, Yuki Nakata, Kandai Nozu, Kazumichi Fujioka

**Affiliations:** Department of Pediatrics, Kobe University Graduate School of Medicine, Kobe, Japan

## Abstract

**Background:**

Meconium peritonitis is a noninfectious chemical peritonitis that occurs following fetal intestinal perforation and leakage of meconium into the abdominal cavity. Because of the lack of appropriate animal models, its pathophysiology has not yet been elucidated. We aimed to create a neonatal mouse model of meconium peritonitis using human meconium slurry (MS).

**Methods:**

A stock MS solution prepared from fresh meconium obtained from healthy term infants was administered intraperitoneally to 4-d-old newborn mice. An MS LD_40_ was then administered, and changes in body weight, hematology, serum biochemistry, and immunomodulatory gene expression were determined. The MS was subjected to antibiotic treatment and heat inactivation to validate the content. Finally, comparisons with nonsurgical neonatal sepsis mouse models were performed.

**Results:**

Dose-dependent mortality rates were observed, with an LD_40_ of 200 µL/body weight established. Substantial hematological and hepatorenal abnormalities and increased inflammatory gene expression were observed. Although antibiotic treatment was ineffective, the survival rate was improved by enzymatic inactivation of MS. Importantly, the systemic responses to MS were distinct from those observed in neonatal sepsis model mice.

**Conclusion:**

The MS model closely reflects the pathology of human neonatal meconium peritonitis and maybe useful in research elucidating the pathophysiology of this condition.

**Impact:**

- In this study, we generated a neonatal mouse model of meconium peritonitis through intraperitoneal administration of human meconium slurry.
- We clarified that the pathogenic agent in meconium slurry is mainly a digestive enzyme, and that the systemic responses elicited by meconium slurry were distinct from those in a neonatal sepsis mouse model.
- As our mouse model is simple and highly reproducible, it is useful for elucidating the pathophysiology of meconium peritonitis.

## Introduction

Meconium peritonitis is a non-infectious chemical peritonitis that occurs following fetal intestinal perforation and leakage of meconium into the abdominal cavity.^1,2^ It has a reported incidence of 1 in 35,000 live births.^3^ The clinical features are diverse but grouped into three types: generalized, cystic, and fibroadhesive.^4^ Meconium peritonitis is a life-threatening condition in the perinatal period,^1^ and, despite recent advances in perinatal management, the mortality rate remains 10–15%.^5^ As for prenatal treatment, although a report suggested the efficacy of fetal ascites drainage, the efficacy of this approach has not been established.^6^ For postnatal treatment, no effective medical or surgical treatment method has been established to date.^2,3^ Therefore, for the development of an effective therapy, elucidation of the pathophysiology of meconium peritonitis is urgently needed. As the meconium is a sterile stool containing eliminated mucosal epithelia, water, bile, bile acids, epithelium cells, and other lipids swallowed with amniotic fluid,^7^ sterile inflammation induced by digestive enzymes contained in the meconium has been assumed; however, only a few studies on meconium content have been reported to date. Lally et al. reported that meconium stimulation results in a marked proinflammatory response by peritoneal macrophages in an in vitro study using adult mouse macrophages.^8^ In clinical observations, Kanamori et al. reported massive elevation of interleukin (IL)-6 and -8 in the ascites of infants with meconium peritonitis, suggesting that inhibition of specific components of the inflammatory response may be therapeutic targets.^9^

However, research on meconium peritonitis pathogenesis has not progressed sufficiently, mainly because of the lack of adequate animal models that accurately reflect the pathology. When considering the generation of a mouse model of meconium peritonitis, it is important to recognize that prenatal intestinal immaturity might play an important role in the onset of meconium peritonitis, as premature infants are prone to intestinal inflammation.^10^ In addition, intestinal tract development in mice differs greatly from that in humans; at birth, it corresponds to 12 weeks of human embryonic development; at 2 weeks of age, it corresponds to 24 weeks of gestation; and at 4 weeks, it corresponds to full-term gestation in humans.^11^ Thus, an animal model using neonatal mice may well mimic intraperitoneal pathology during the early fetal period in humans. However, the development of a mouse model of meconium peritonitis presents two difficulties. One is that mouse fetuses are too small to undergo surgery to perforate intestines and induce meconium leakage, and the other is that intraperitoneal implantation of the human meconium is too invasive for tiny newborn pups. Therefore, we decided to use a non-surgical method to develop a preterm sepsis mouse model in 4-d-old newborn mice, which was adapted from the cecal slurry (CS) model described by Wynn et al.^12,13^. Briefly, CS is prepared from a suspension of cecal contents removed from adult mice and administered intraperitoneally to mouse pups, resulting in septic peritonitis. In addition, we combined the protocol described by Starr et al. ^14^ which facilitates long-term storage of a stock CS preparation to ensure consistency of bacterial content. As it is impossible to collect enough meconium from mouse fetuses for experiments, we planned to implant a human meconium suspension intraperitonially in mice to create a neonatal meconium peritonitis mouse model. For human-mouse fecal transplantation experiments, we referred to the mouse lethality assay, which is an established diagnostic method for botulism.^15^

In this study, we created a meconium peritonitis mouse model using a technique previously used for a non-surgical preterm sepsis mouse model with intraperitoneal administration of a human meconium suspension into 4-d-old mice.

## Methods

### Animals

Adult FVB/NJcl mouse breeders were obtained from CLEA Japan, Inc. (Tokyo, Japan), fed a standard rodent diet, and provided access to water *ad libitum*. All pups were kept with their mothers throughout the study period. Pups were randomized on an individual basis within each litter for each experiment. To eliminate litter bias, at least three different litters were used for each experimental group. For identification, the back of each pup was labeled using a Sharpie marker. This study was approved by the Kobe University Institutional Animal Care and Use Committee (protocol P170103).

### Stock human meconium slurry (MS) preparation

**Fig. 1:**
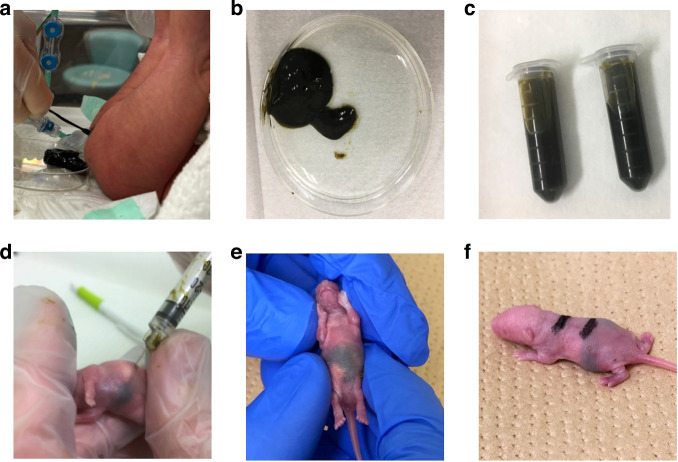
Preparation of meconium slurry (MS) stock and meconium peritonitis induction. **a**, **b** Fresh meconium aseptically collected from healthy term newborns. **c** Meconium contents were mixed with 1.0 mL of phosphate-buffered saline (PBS) per 500 mg of meconium (500 mg/mL). **d** Four-day-old mouse pups were administered various doses of MS intraperitoneally. **e**, **f** Appearance of mouse pups right after intraperitoneal administration of 200 µL of MS.

After obtaining parental consent, fresh meconium from healthy term human newborns was collected aseptically via rectal stimulation. A total of six infants provided sterile meconium, and the median meconium weight was 4.69 g (minimum 1.95 g, maximum 10.78 g). Because sterility could not be determined at the time of MS preparation, all meconium was used without pooling. The collected meconium contents were weighed, and 1.0 mL of phosphate-buffered saline (PBS) per 500 mg of meconium was added (500 mg/mL, Fig. 1). After stirring the meconium and PBS mixture vigorously, each MS sample was aspirated with a syringe through a 23-gauge needle. Then, to create stocks, MS samples were transferred to cryovials in 1-or 2-mL aliquots and stored at −80 °C. Before experimental use, an aliquot of stock MS was thawed at room temperature, and 50 µL was plated onto 1.5% agar containing brain/heart infusion (BHI) broth.^14^ Agar plates were then incubated at 37 °C for 24 h, and colony forming units (CFUs) were counted. Sterile stocks without bacterial growth were used in all studies. For each study, an MS aliquot was thawed and vortexed before injection.

### Meconium peritonitis induction

To induce meconium peritonitis, 4-d-old mouse pups, which are immunologically equivalent to human preterm infants,^13,16^ were administered various doses (100–300 µL) of MS intraperitoneally and then closely monitored daily for health and survival for up to 7 d, based on a previously reported method.^14^ To monitor meconium peritonitis development, body weights of survivors were recorded daily for 7 d beginning 24 h after MS administration. In this dose-dependent experiment, different MS stocks collected from three infants were used to eliminate the influence of differences in meconium content among individual donors and to confirm reproducibility. Each subsequent experiment was performed with an MS stock prepared from a single infant.

### Antibiotic treatment

To determine the protective efficacy of antibiotics in this MS model, we randomly administered 100 mg/kg of imipenem and cilastatin sodium (IPM-MS) or an equivalent volume of normal saline (Veh-MS) subcutaneously to 4-d-old mice immediately before meconium peritonitis induction, based on a method used in a previous study.^17^ Right after IPM administration, meconium peritonitis was induced via intraperitoneal administration of 200 µL of MS and then, survival was monitored for 7 d.

### Heat inactivation of MS

To determine the effects of mortality of digestive enzyme inactivation via heat-shock pretreatment of MS in this model, we randomly administered 200 µL of non-treated (MS) or heat-shocked (70 °C [HS70-MS] or 100 °C [HS100-MS] for 15 min) MS intraperitoneally to 4-d-old mice and then survival was monitored for 7 d.

### Hematology and biochemistry

At 24 h (i.e., 5 d of life) post-meconium peritonitis induction, pups were sacrificed via decapitation under room air, and 30–80 µL of blood pooled from two or three pups was immediately collected in capillary blood collection tubes containing lithium heparin (Capiject; Terumo Medical Corporation, Tokyo, Japan) for complete blood counts (CBCs) using an automated differential method and biochemistry. CBC measurements were performed using a veterinary hematology analyzer (Thinka CB-1010, ARKRAY, Kyoto, Japan), and biochemical measurements were performed at the Diagnostic Laboratory of Cosmo Bio (Tokyo, Japan).

### Polymerase chain reaction (PCR) arrays

At 6 h post-meconium peritonitis induction, pups were sacrificed, and 5 × 5 × 1-mm pieces of liver were immediately placed in liquid nitrogen and stored at −80 °C until use. Total RNA was extracted according to standard laboratory procedures using an RNAeasy Mini Kit (Qiagen, Valencia, CA). cDNA was synthesized using the RT2 First Strand Kit (Qiagen, Hilden, Germany). PCR array kits (Qiagen, Hilden, Germany) that screened for 84 genes were used to analyze mouse innate and adaptive immune responses (catalog no. PAMM-052Z). Real-time PCR was performed using RT2 Real-Time SYBR Green/ROX PCR Master Mix (Qiagen) on an Applied Biosystems (Foster City, CA) 7500 FAST Real-Time PCR System (Thermo Fisher Scientific). Fold changes in gene expression levels in MS or CS over vehicle-treated control levels, as well as MS over CS levels, were calculated using the ΔΔCt method as previously described.^13,16^

### Sepsis induction

A non-surgical neonatal sepsis mouse model that we reported previously was adopted to compare the pathologies of meconium peritonitis and sepsis.^13,16,18^ In brief, to create stock CS solutions, adult wild-type FVB mice were sacrificed and whole cecums were harvested. Cecal contents were collected, pooled, weighed, and then mixed with 0.5 mL of sterile water per 100 mg of cecal content. The mixture was filtered through a 100-µm mesh strainer, added to an equal volume of 30% glycerol in PBS, and then transferred to cryovials as CS stock in 1-mL aliquots for storage at −80 °C.^13^ To induce sepsis, 4-d-old mouse pups were administered a CS dose of LD_60_ (1.5 mg/g bw) for sepsis induction, based on our previous studies.　Using this CS model, hematology, biochemistry, and PCR experiments were conducted simultaneously with those in the MS model.

### Statistical analyses

Statistical analyses were performed using the log-rank test for survival curves, unpaired Student’s two-tailed *t*-test for PCR array analyses, Mann–Whitney test or chi-square test for comparisons of two groups, and Kruskal-Wallis test with Dunn’s multiple comparison test for comparisons of three groups or more. Differences were considered statistically significant at *P* < 0.05. Data are expressed as means ± standard deviations.

## Results

### Non-surgical meconium peritonitis model using human meconium slurry (MS)

#### Dose-dependent effects

**Fig. 2:**
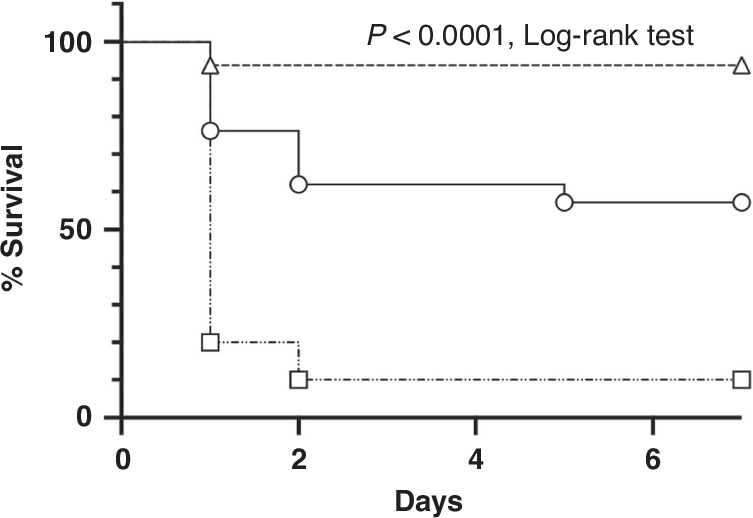
Survival curves. Four-day-old wild-type pups after intraperitoneal injection of meconium slurry at various doses: 100 (▵, *n* = 32), 200 (○, *n* = 21), and 300 (□, *n* = 10) µL. Significant dose-dependent mortality was observed with an LD_40_ of 200 µL (*P* < 0.0001, log-rank test).

Similar to previous findings in our sepsis model, a marked dose-dependent increase in mortality (6% for 100 µL of MS, 43% for 200 µL of MS, and 90% for 300 µL of MS, *n* ≥ 10 in each group, *P* < 0.0001, log-rank test, Fig. 2) was observed.

#### Body weight change post-meconium peritonitis induction

**Fig. 3:**
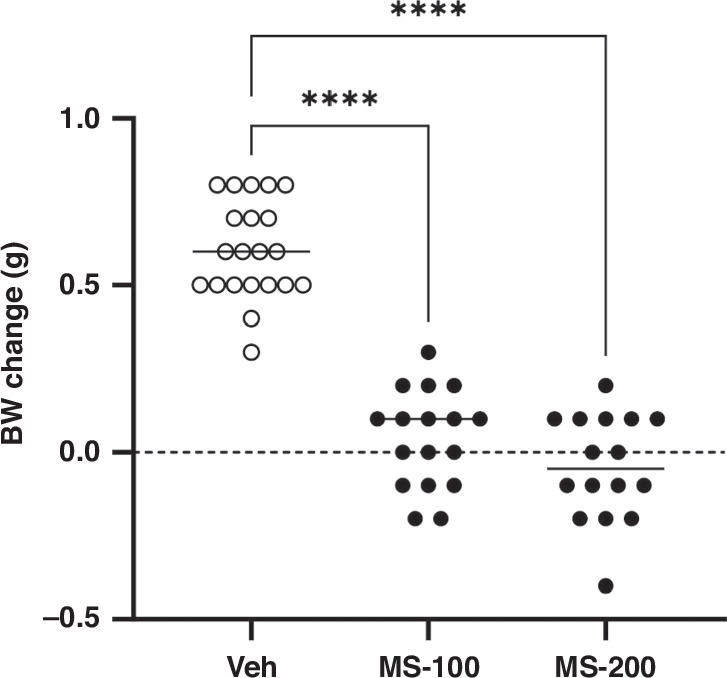
Body weight gain at 24 h after sepsis induction. Body weight gain is shown for surviving pups only: vehicle-treated group (Veh: ○, 0.6 ± 0.1 g, *n* = 21), 100 µL meconium slurry (MS)-treated group (MS-100: ●, 0.0 ± 0.1 g, *n* = 17), and 200 µL MS-treated group (MS-200: ●, 0.0 ± 0.2 g, *n* = 16). Body weight changes in the MS-100 and MS-200 groups were significantly lower than those of the Veh groups ****P < 0.0001.

We then compared body weight gains at 24 h post-meconium peritonitis induction in surviving pups in the normal saline (Veh), MS-100, and MS-200 groups. Body weight changes could not be examined in the MS-300 group because most pups died within 24 h post-meconium peritonitis induction. When comparing body weight gains at 24 h post-meconium peritonitis induction, no significant difference was noted between the MS-100 (0.0 ± 0.1 g, *n* = 17) and MS-200 (0.0 ± 0.2 g, *n* = 16) groups. However, the body weight gains in these groups was significantly lower than those in the Veh group (0.6 ± 0.1 g, *n* = 21, *P* < 0.0001, Fig. 3). In the experiment that followed, we administered 200 µL of MS (LD_40_) intraperitoneally to 4-d-old newborn pups.

#### Effect of antibiotic pretreatment of MS

**Fig. 4:**
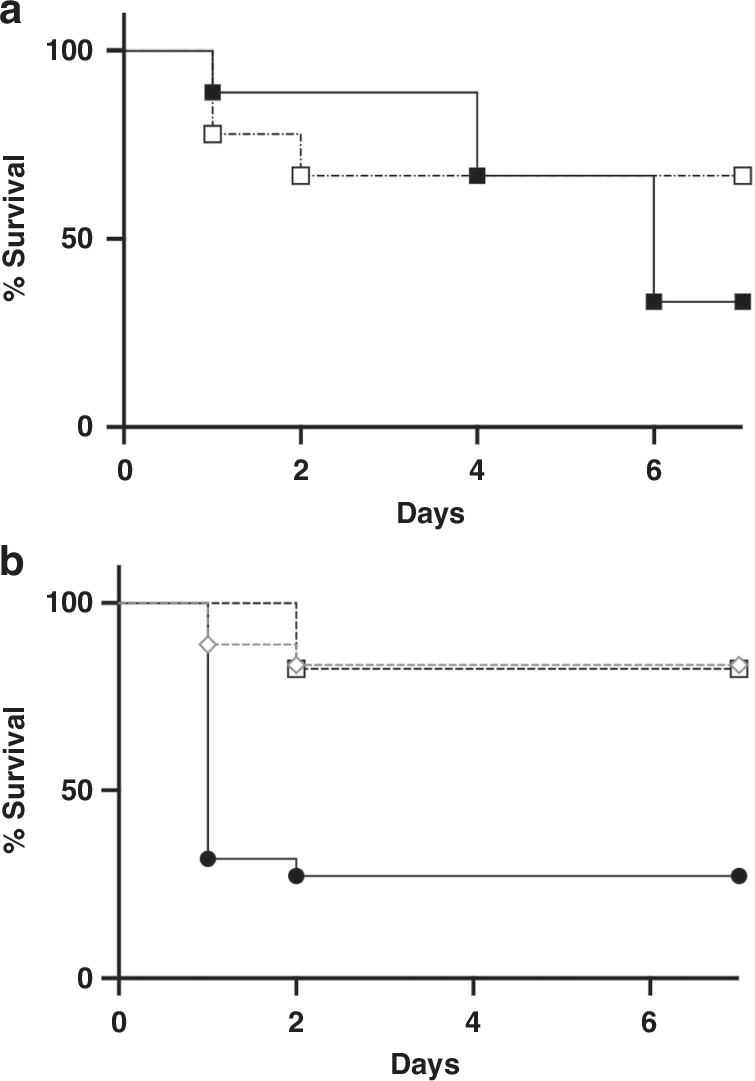
Survival curves. **a** Kaplan-Meier survival plots of 4-d-old pups subcutaneously treated with IPM (IPM-MS: ■, *n* = 9) or an equivalent volume of normal saline (Veh-MS: □, *n* = 9) immediately before meconium peritonitis induction. The mortality rate was 67% in the IPM-MS group and 33% in the Veh-MS group; this difference was not significant (*P* = 0.54). **b** Kaplan-Meier survival plots of 4-d-old pups treated with meconium slurry (MS) (●, MS, *n* = 22), 70 °C heat-shocked MS (♢, HS70-MS, *n* = 18), and 100 °C heat-shocked MS (□, HS100-MS, *n* = 17). The mortality rates following the administration of HS70-MS (17%) and HS100-MS (18%) were significantly lower than those following the administration of MS (73%) (*P* < 0.0001, log-rank test).

When we compared mortality rates in the IPM-MS (*n* = 9) and Veh-MS (n = 9) groups, we found that the mortality rate was 67% in the IPM-MS group and 33% in the Veh-MS group, with no significant difference between them (*P* = 0.54, Fig. 4a).

#### Effect of heat-inactivation pretreatment of MS

When we compared the mortality rates in the three groups, we found that heat inactivation of MS significantly reduced mortality from 73% (MS, *n* = 22) to 17% (HS70-MS, *n* = 18) and 18% (HS100-MS, *n* = 17) (*P* < 0.0001; Fig. 4b).

#### Hematological changes post-meconium peritonitis induction

**Fig. 5:**
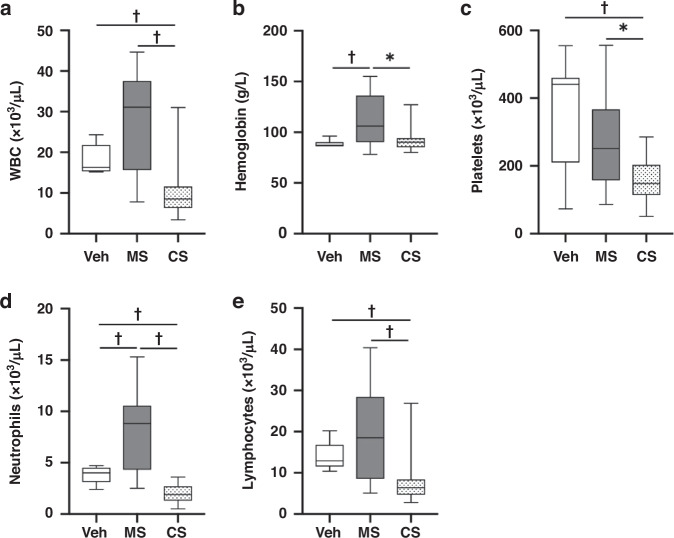
Hematological changes. **a** White blood cell (10^3^ cells/mL), (**b**) hemoglobin levels (g/L), (**c**) platelets (10^3^ cells/mL), (**d**) neutrophils (10^3^ cells/mL), and (**e**) lymphocytes (10^3^ cells/mL) of controls pups (Veh, *n* = 11), meconium peritonitis-induced pups 24 h after MS administration (MS, *n* = 13), and sepsis-induced pups 24 h after CS administration (CS, *n* = 15), †*P* < 0.01, *P < 0.05.

Twenty-four hours post-meconium peritonitis induction, hemoglobin (Hgb) counts were found to be significantly higher in MS pups (112.2 ± 25.5 g/dL, *n* = 13) than in Veh pups (88.5 ± 3.5 g/dL, *n* = 11, *P* < 0.01). White blood cells in MS pups (27,320 ± 12,150 /µL, *n* = 13) were higher than those in Veh pups (18540 ± 3719 /µL), and platelet counts in MS pups (272.9 ± 136 × 10^3^/µL, *n* = 13) were lower than those in Veh pups (357.2 ± 150.7 × 10^3^/µL, *n* = 11), but these differences were not statistically significant (*P* = 0.10 and 0.12, respectively). When we compared leukocyte fractions, only neutrophils were significantly higher in MS pups (8054 ± 383/μL, *n* = 13) than in Veh pups (3836 ± 769/μL, *n* = 11, *P* < 0.005, Fig. 5).

#### Serum biochemistry post-meconium peritonitis induction

**Fig. 6:**
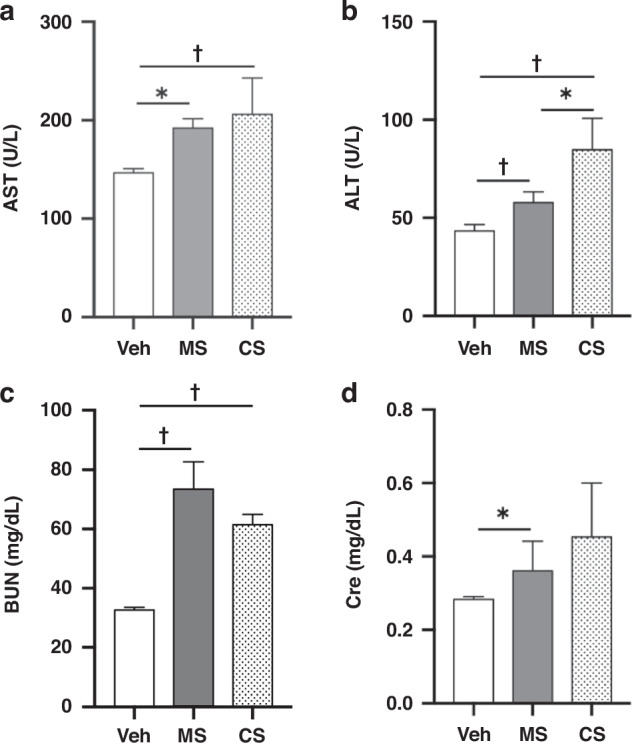
Serum biochemistry. **a** Serum AST levels (U/L), (**b**) ALT levels (U/L), (**c**) BUN levels (mg/dL), and (**d**) Cre levels (mg/dL) of control pups (Veh, *n* = 6), meconium peritonitis-induced pups 24 h after MS administration (MS, *n* = 6), and sepsis-induced pups 24 h after CS administration (CS, *n* = 4), † *P* < 0.01, *P < 0.05. AST serum aspartate aminotransaminase, ALT alanine aminotransferase, BUN blood urea nitrogen, Cre creatinine.

To determine the effects of meconium peritonitis induction in livers and kidneys, serum AST/ALT and BUN/Cre levels were measured 24 h post-meconium peritonitis induction. AST (193 ± 9 U/L, *n* = 6) and ALT (58 ± 5 U/L, *n* = 6) levels in MS pups were significantly higher than those in Veh pups (147 ± 4 U/L and 44 ± 3 U/L, *n* = 6, *P* = 0.02 and <0.005, respectively). In addition, BUN (74 ± 9 mg/dL, *n* = 6) and Cre (0.36 ± 0.08 mg/dL, *n* = 6) levels in MS pups were significantly higher than those in Veh pups (33 ± 1 mg/dL and 0.29 ± 0.01 mg/dL, *n* = 6, *P* < 0.005 and 0.03, respectively, Fig. 6).

#### Expression profiles of genes involved in innate and adaptive immunity post-meconium peritonitis induction

To determine the expression levels of genes involved in innate and adaptive immune responses, we isolated RNA from livers harvested 6 h post-meconium peritonitis induction. Using real-time quantitative PCR, we found that the transcripts of three genes were significantly upregulated in the livers of MS mice compared to those in Veh mouse livers (fold change >4.0, *P* < 0.05, Table 1). The upregulated genes were Cd14 (128.2-fold), Cxcl10 (10.8-fold), and Il10 (5.7-fold).

**Table 1:** Fold changes in expression levels of immune genes in MS and CS groups compared to controls.

	MS		CS	
Gene symbol	Fold change	*P*	Fold change	*P*
Ccl5	1.34	0.375	**4.17**	**0.001**
CD14	**128.19**	**0.027**	**582.68**	**<0.001**
CXCL10	**10.81**	**0.011**	**9.67**	**0.004**
Cxcr3	−1.37	0.455	**–4.99**	**0.034**
Icam1	1.07	0.680	**4.14**	**<0.001**
Il10	**5.67**	**0.036**	**15.98**	**<0.001**
Il1b	1.79	0.083	**13.02**	**0.002**
Il1r1	1.30	0.353	**6.97**	**<0.001**
Myd88	2.58	0.062	**4.26**	**<0.001**
Nfkbia	3.48	0.038	**6.80**	**<0.001**
Tlr2	−1.14	0.746	**8.10**	**0.001**
Tlr5	−1.72	0.247	**-11.65**	**0.014**

Fold change was calculated as the gene expression levels in the MS group (*n* = 3) and CS group (*n* = 3) pups compared to that in vehicle-treated control group pups. MS, meconium peritonitis-induced pups 6 h after meconium slurry administration; CS, sepsis-induced pups 6 h after cecal slurry administration.

### Comparison to non-surgical neonatal sepsis mouse model

#### Hematological changes post-meconium peritonitis induction

Twenty-four hours post-induction, white blood cell, hemoglobin, and platelet counts in MS pups (27320 ± 12150/µL, 112.2 ± 25.5 g/dL, and 272.9 ± 136 × 10^3^/µL, respectively, *n* = 13) were significantly higher than those in CS pups (10020 ± 6620/µL, 91.9 ± 12.0 g/dL, and 157.2 ± 58 × 10^3^/µL and *P* < 0.0001, *P* = 0.03, and *P* = 0.01, respectively, *n* = 15). When we compared leukocyte fractions, neutrophils and lymphocytes were significantly higher in MS pups (8054 ± 383/μL and 18,577 ± 1122/μL, respectively, *n* = 13) than in CS pups (1927 ± 586/μL and 7780 ± 598/μL and *P* < 0.0001 and *P* < 0.005, respectively, *n* = 15, Fig. 5).

#### Serum biochemistry post-meconium peritonitis induction

Twenty-four hours post-induction, ALT levels in MS pups were significantly lower than those in CS pups (58 ± 5 U/L, *n* = 6 vs. 85 ± 16 U/L, *n* = 4, *P* = 0.02), but no significant difference was found in AST levels (193 ± 9 U/L, *n* = 6 vs. 207 ± 36 U/L, *n* = 4, *P* = 0.64) between the two groups. For renal function, no significant differences were found in BUN (74 ± 9 mg/dL, *n* = 6 vs. 62 ± 3 mg/dL, *n* = 4, *P* = 0.07) or Cre (0.36 ± 0.08 mg/dL, *n* = 6 vs. 0.46 ± 0.15 U/L, *n* = 4, *P* = 0.39) levels between the groups (Fig. 6).

#### Expression profiles of genes involved in innate and adaptive immunity post-meconium peritonitis and post-sepsis induction

On comparing the expression levels of genes involved in innate and adaptive immune responses between MS and CS mice at 6 h post-induction, transcripts of 14 genes were found to be significantly decreased in the livers of MS mice compared to those of CS mice (fold change >2.0, *P* < 0.05, Table 2). The only upregulated gene was *Tlr5* (6.76-fold increase, *P* < 0.05).

**Table 2:** Fold changes in expression levels of immune genes in MS groups compared to CS group.

	MS	
Gene symbol	Fold change	*P*
C5ar1	−2.25	0.013
CCL12	−4.11	0.012
Ccl5	−3.12	0.002
CD14	−4.55	0.001
Crp	−3.79	<0.001
Icam1	−3.85	0.001
Il10	−2.82	0.011
Il1a	−3.2	0.047
Il1b	−7.27	0.003
Il1r1	−5.36	<0.001
Irf7	−2.93	0.040
Slc11a1	−2.06	0.038
Tlr2	−9.21	0.001
Tlr5	6.76	0.049
Tnf	−3.66	0.003

Fold change was calculated as the expression levels of the MS group (*n* = 3) over the CS group pups (*n* = 3). MS, meconium peritonitis-induced pups 6 h after meconium slurry administration; CS, sepsis-induced pups 6 h after cecal slurry administration.

## Discussion

In this study, we used human neonatal meconium suspensions to create a nonsurgical neonatal meconium peritonitis mouse model. This MS model was associated with hematologic abnormalities, hepatorenal dysfunction, changes in liver inflammatory gene expression, and the inability of antibiotics, but ability of heat-treated MS, to improve survival. We compared this model with a neonatal sepsis model established using the cecal slurry method and found that the two models exhibited different phenotypes.

We established a meconium peritonitis mouse model via intraperitoneal administration of a suspension of fresh meconium from human neonates to neonatal mice. To create the meconium peritonitis model in premature neonatal pups, we modified the non-surgical sepsis induction technique established by Wynn et al. This technique involves intraperitoneal administration of adult cecal contents suspended in dextrose.^12^ Non-invasive administration was achieved by suspending fresh meconium. Regarding the experimental validity of heterologous administration of human meconium to mice, we considered it appropriate because the mouse lethality assay is used as a standard procedure for clinical detection of botulinum toxin, in which human fecal contents are administered intraperitoneally to laboratory mice.^15^ However, completely excluding the influence of a heterologous immune response to residual human tissues contained in MS is difficult. This represents a limitation of the model. There have been several reports of adult animal studies investigating the intestinal damage induced by meconium in amniotic fluid in gastroschises. Akgur et al. injected human neonatal urine or a 5% human meconium suspension intraperitoneally into adult rats twice a day for 5 d and observed histological changes in the intestinal mucosa. Intraperitoneal injection of neonatal human diluted meconium caused intestinal damage in adult rat intestines, including serosal thickening, inflammation, and focal fibrin and collagen deposits, whereas neonatal human urine did not have any effect.^19^ In contrast, Samala et al. performed a similar experiment but found maximum bowel damage in the urine group, with a lesser degree of damage observed in the meconium group as mild lymphoid hyperplasia.^20^ However, these studies examined the effects of meconium contained in amniotic fluid on the intestine, and therefore, did not mimic meconium peritonitis pathogenesis, in which meconium itself leaks into the abdominal cavity and provokes a systemic reaction. We succeeded in creating a systemic inflammatory model that mimics meconium peritonitis by administering a single dose of high concentrations of MS. Using a meconium peritonitis animal model created by intraperitoneal injection of high-concentration meconium similar to ours, Tokar et al. reported a rat model administered a 40% solution of meconium into peritoneal cavities for 3 d, which resulted in macroscopically apparent thickening and dilatation of intestinal loops.^21^ However, as their experiments were conducted using adult rats, not fetuses or neonates that are affected by meconium peritonitis, investigations of systemic reactions are also lacking. Therefore, we believe that the model proposed in this study is the first neonatal mouse model that accurately recapitulates the meconium peritonitis phenotype.

In our MS model, the survival rate did not improve with antibiotic treatment; however, it improved when heat-shock-treated MS was used to induce peritonitis. As antibiotic administration did not contribute to improved survival, the main pathology of this model is unlikely to be due to bacterial infection, unlike sepsis. Rather, meconium contains various digestive enzymes,^2^ and when MS is heat-shocked, these enzymes are inactivated owing to thermal denaturation. Therefore, the present results, in which mortality was reduced with heat-shocked MS, suggest that digestive enzymes contained in the meconium may have a strong influence on the pathology of this meconium peritonitis model.

In addition, compared to the sepsis model, the MS model showed different hematological and biochemical results, with white blood cell, hemoglobin, and platelet parameters all significantly higher. Neonatal sepsis is known to cause bone marrow suppression, including leukopenia, neutropenia, and thrombocytopenia,^22,23^, but reactive hematopoiesis may be enhanced in the MS model. Based on the biochemical analysis, the MS model exhibited impaired hepatic and renal functions compared to the control group, suggesting multiple organ damage. ALT values were significantly lower in the MS than CS model, suggesting that the degree of organ damage in the MS model was lower than that in the sepsis model. Regarding liver inflammatory markers, compared with the control group, the MS model showed an increase in *Cd14*, *Cxcl10*, and *Il10* expression. However, compared to the CS model, the MS model showed increased *Tlr5* expression but decreased expression of many immune-inflammatory genes. When interpreting this, we assumed that CS solutions used in the CS model contain mouse-derived digestive enzymes; therefore, it is necessary to consider the possibility that digestive enzymes also modified the inflammatory response in that model. In addition, another type of neonatal sepsis model generated via intravenous injection of *Staphylococcus epidermidis* showed more diversity in effects on the Toll-like receptor transcriptome in the liver, with increases in expression of more than the three genes increased in the MS model.^24^ Thus, this MS model is a more limited inflammatory response model than the neonatal sepsis model. Based on the results described above, we believe the MS model presented here is a specific animal model that is substantially different from the sepsis model in terms of systemic organ damage and immune responses.

Few studies have examined the pathophysiology of meconium peritonitis in clinical settings. In an in vitro experiment, Lally et al. showed that when peritoneal macrophages harvested from adult mice were stimulated with sterile human meconium, tumor necrosis factor-alpha (TNF-α) and procoagulant activity (PCA) increased. They also reported an increase in TNF mRNA in a human meconium concentration-dependent manner, suggesting that the TNF response might be mediated at the pre-transcriptional level. In addition, they revealed that the increase in PCA was limited to <5% by protein kinase C (PKC) inhibition, suggesting that the PCA response is regulated by PKC as much as it is with lipopolysaccharide.^8^ Additionally, a case report of meconium peritonitis suggested that the observed coagulopathy was due to a systemic inflammatory response during the fetal period.^25^ Collectively, these studies suggest that proinflammatory responses and procoagulant activity may contribute to the pathology of meconium peritonitis. In another study, Kanamori et al. measured cytokines and chemokines in the serum and ascites of patients with meconium peritonitis and found IL-6 and IL-8 levels increased in all cases. They also reported that cystic fluid drainage did not completely suppress this inflammation. They concluded that patients with meconium peritonitis exhibit typical clinical symptoms of fetal inflammatory response syndrome and that IL-6 and IL-8 play important roles in the pathogenesis of this condition.^9^

This study has some limitations. First, meconium peritonitis is a condition in which the meconium leaks into the abdominal cavity after fetal gastrointestinal perforation; however, our MS model is not based on gastrointestinal perforation. Newborn pups are so fragile that they cannot withstand surgical procedures such as gastrointestinal perforation.^13^ Thus, we created a minimally invasive model that mimics the pathological conditions caused by meconium leakage into the abdominal cavity of newborns. Based on the experimental results, this model sufficiently mimics the conditions of human meconium peritonitis. To create a meconium peritonitis model that demonstrates the effects of gastrointestinal perforation, a combination of surgical procedures and MS administration in large animals is necessary. Second, due to the nature of meconium, the prepared MS was extremely viscous and fine adjustment of the dosage was difficult; thus, the dosage was adjusted in 100-µL increments. Therefore, the dose per body weight could not be precisely adjusted in each group in this study, as was performed in our previous studies.^13,18^ However, as this model clearly showed a dose-dependent increase in mortality rate, we consider it sufficient as a model for pathophysiological analysis. Third, the composition of digestive enzymes in MS was not investigated. Meconium contains various digestive components and enzymes such as trypsin,^26,27^, and differences in these enzymes exist among individuals.^28^ Such individual variation could underlie the differences in peritonitis severity observed with the use of different MS stocks. To reduce the influence of differences in meconium composition on experimental results, MS from the same source was used in each experiment, except for the dose-dependent experiments.

In conclusion, we established a mouse model of meconium peritonitis via intraperitoneal administration of a suspension of fresh meconium from human neonates into neonatal mice. The MS model exhibits non-infectious systemic inflammation, in addition to hematological and hepatorenal abnormalities. While antibiotic treatment was ineffective, the survival rate of MS model mice was improved by enzymatic inactivation using heat shock. This model reflects the pathology of human neonatal meconium peritonitis. As our mouse model is simple and highly reproducible, it can be used in research to elucidate the pathophysiology of meconium peritonitis.

## Data Availability

The data that support the findings of this study are available upon reasonable request.

## References

[1] Nam, S. H. et al. Experience with meconium peritonitis. *J. Pediatr. Surg.* **42**, 1822–1825 (2007). 18022430 10.1016/j.jpedsurg.2007.07.006

[2] Uchida, K. et al. Meconium peritonitis: Prenatal diagnosis of a rare entity and postnatal management. *Intractable Rare Dis. Res.* **4**, 93–97 (2015). 25984428 10.5582/irdr.2015.01011 PMC4428193

[3] Jiang, Y. et al. Can early surgery improve the outcome of patients with meconium peritonitis? a single-center experience over 16 years. *BMC Pediatr.* **19**, 473 (2019). 31795969 10.1186/s12887-019-1844-5 PMC6889670

[4] Lorimer, W. S. Jr. & Ellis, D. G. Meconium peritonitis. *Surgery* **60**, 470–475 (1966). 5920370

[5] Wang, C. N. et al. Meconium peritonitis in utero-the value of prenatal diagnosis in determining neonatal outcome. *Taiwan J. Obstet. Gynecol.* **47**, 391–396 (2008). 19126503 10.1016/S1028-4559(09)60004-8

[6] Okawa, T., Soeda, S., Watanabe, T., Sato, K. & Sato, A. Repeated paracentesis in a fetus with meconium peritonitis with massive ascites: A case report. *Fetal Diagn. Ther.* **24**, 99–102 (2008). 18648207 10.1159/000142136

[7] Trdin, A. et al. Mercury speciation in meconium and associated factors. *Environ. Res*. **179**, 108724 (2019). 31627028 10.1016/j.envres.2019.108724

[8] Lally, K. P., Mehall, J. R., Xue, H. & Thompson, J. Meconium stimulates a pro-inflammatory response in peritoneal macrophages: implications for meconium peritonitis. *J. Pediatr. Surg.* **34**, 214–217 (1999). 10022175 10.1016/s0022-3468(99)90260-9

[9] Kanamori, Y. et al. Interleukin 6 and interleukin 8 play important roles in systemic inflammatory response syndrome of meconium peritonitis. *Surg. Today* **42**, 431–434 (2012). 22068677 10.1007/s00595-011-0034-3

[10] Humberg, A. et al. Preterm birth and sustained inflammation: Consequences for the neonate. *Semin Immunopathol.* **42**, 451–468 (2020). 32661735 10.1007/s00281-020-00803-2 PMC7508934

[11] McElroy, S. J. & Weitkamp, J. H. Innate immunity in the small intestine of the preterm infant. *Neoreviews* **12**, e517–e526 (2011). 22639551 10.1542/neo.12-9-e517 PMC3359837

[12] Wynn, J. L. et al. Increased mortality and altered immunity in neonatal sepsis produced by generalized peritonitis. *Shock* **28**, 675–683 (2007). 17621256 10.1097/SHK.0b013e3180556d09

[13] Fujioka, K. et al. Induction of heme oxygenase-1 attenuates the severity of sepsis in a non-surgical preterm mouse model. *Shock* **47**, 242–250 (2017). 27454382 10.1097/SHK.0000000000000689

[14] Starr, M. E. et al. A new cecal slurry preparation protocol with improved long-term reproducibility for animal models of sepsis. *PLoS One* **9**, e115705 (2014). 25531402 10.1371/journal.pone.0115705 PMC4274114

[15] Lindstrom, M. & Korkeala, H. Laboratory diagnostics of botulism. *Clin. Microbiol Rev.* **19**, 298–314 (2006). 16614251 10.1128/CMR.19.2.298-314.2006 PMC1471988

[16] Fujioka, K., Kalish, F., Zhao, H., Wong, R. J. & Stevenson, D. K. Heme oxygenase-1 deficiency promotes severity of sepsis in a non-surgical preterm mouse model. *Pediatr. Res* **84**, 139–145 (2018). 29795214 10.1038/s41390-018-0028-6

[17] Craciun, F. L. et al. Early murine polymicrobial sepsis predominantly causes renal injury. *Shock* **41**, 97–103 (2014). 24300829 10.1097/SHK.0000000000000073 PMC5547441

[18] Ashina, M. et al. Recombinant human thrombomodulin attenuated sepsis severity in a non-surgical preterm mouse model. *Sci. Rep.* **10**, 333 (2020). 31941991 10.1038/s41598-019-57265-2 PMC6962223

[19] Akgur, F. M., Ozdemir, T., Olguner, M., Aktug, T. & Ozer, E. An experimental study investigating the effects of intraperitoneal human neonatal urine and meconium on rat intestines. *Res Exp. Med. (Berl.)* **198**, 207–213 (1998). 9879599 10.1007/s004330050104

[20] Samala, D. S. et al. To observe the intensity of the inflammatory reaction caused by neonatal urine and meconium on the intestinal wall of rats in order to understand etiology of intestinal damage in gastroschisis. *J. Indian Assoc. Pediatr. Surg.* **19**, 5–9 (2014). 24604977 10.4103/0971-9261.125944 PMC3935304

[21] Tokar, B. et al. The effect of hyperbaric oxygen treatment on the inflammatory changes caused by intraperitoneal meconium. *Pediatr. Surg. Int* **19**, 673–676 (2003). 14566418 10.1007/s00383-003-1036-z

[22] Hornik, C. P. et al. Use of the complete blood cell count in early-onset neonatal sepsis. *Pediatr. Infect. Dis. J.* **31**, 799–802 (2012). 22531231 10.1097/INF.0b013e318256905c PMC3399972

[23] Chauhan, N., Tiwari, S. & Jain, U. Potential biomarkers for effective screening of neonatal sepsis infections: An overview. *Micro. Pathog.* **107**, 234–242 (2017). 10.1016/j.micpath.2017.03.042 28377234

[24] Kronforst, K. D. et al. A neonatal model of intravenous staphylococcus epidermidis infection in mice <24 H old enables characterization of early innate immune responses. *PLoS One* **7**, e43897 (2012). 22970147 10.1371/journal.pone.0043897 PMC3435332

[25] Kobayashi, Y., Nakano, T., Hidaka, N. & Kato, K. A rare case of fetal meconium peritonitis developing coagulopathy in utero. *J. Med Ultrasound* **27**, 205–207 (2019). 31867196 10.4103/JMU.JMU_25_19 PMC6905251

[26] Ivanov, V. A. Meconium aspiration syndrome treatment - new approaches using old drugs. *Med. Hypotheses* **66**, 808–810 (2006). 16364559 10.1016/j.mehy.2005.09.046

[27] Ivanov, V. A., Gewolb, I. H. & Uhal, B. D. A new look at the pathogenesis of the meconium aspiration syndrome: A role for fetal pancreatic proteolytic enzymes in epithelial cell detachment. *Pediatr. Res.* **68**, 221–224 (2010). 20551860 10.1203/PDR.0b013e3181ebd4c3

[28] Naritaka, N. et al. Profile of bile acids in fetal gallbladder and meconium using liquid chromatography-tandem mass spectrometry. *Clin. Chim. Acta* **446**, 76–81 (2015). 25887994 10.1016/j.cca.2015.04.008

